# Factors Associated with Adherence to the Mediterranean Diet and Dietary Habits among University Students in Lebanon

**DOI:** 10.1155/2021/6688462

**Published:** 2021-01-22

**Authors:** Jennifer S. El Hajj, Sofi G. Julien

**Affiliations:** Department of Nutrition and Food Sciences, Faculty of Arts and Sciences, Holy Spirit University of Kaslik, P.O. Box 446, Jounieh, Mount Lebanon, Lebanon

## Abstract

Although the Mediterranean Diet has been acknowledged as the best overall diet for the year 2020, it has seen a decrease in its adherence over the past years. This is due to several reasons, one of which is the gradual shift to a more westernized diet with all the influences that occur especially on university students whose dietary choices set a path for future dietary habits. The aim of this study is to check the level of adherence to the Mediterranean Diet and frequency of breakfast consumption among university students in Lebanon and check whether they are influenced by sociodemographic, anthropometric, dietary knowledge, or academic data. A cross-sectional questionnaire was electronically sent to randomly selected students (210 females and 93 males) from different universities across Lebanon, aged between 18 and 25 years old. The questionnaire was filled online, and all data were self-reported. The Mediterranean Diet Quality Index (KIDMED) was used as a tool to evaluate adherence to the Mediterranean Diet. The results showed that 18.8% of respondents had high adherence to the Mediterranean Diet. Students who reported always consuming breakfast and not skipping meals had significantly higher adherence to the MD. Furthermore, students with lower BMI and higher KIDMED scores had significantly more correct answers on the nutritional knowledge questions. In addition, there was a significant difference in the average KIDMED scores between different GPA categories, most notably when comparing high and poor MD adherence; students with excellent GPA scores had higher adherence to the MD than those with poor GPA scores. In conclusion, nutrition awareness in a university setting is very important since it may positively affect academic outcomes and may be the last chance to teach and engrave healthy eating patterns to a large scale of students.

## 1. Introduction

The Mediterranean Diet (MD) refers to a dietary pattern characterizing the eating habits of populations living in the geographical area of the Mediterranean basin. It was written in 1614 when an Italian writer, Giacomo Castelvetro, tried to socially impact people living in England by introducing the idea of consuming more fresh fruits and vegetables [1]. Scientific evidence of its benefits reached the public when the American scientist Ancel Keys observed a significantly lower rate of coronary heart disease in Southern Italian compared to the American population ascribable to their dietary habits [2]. Albeit evolved over time and with some differences across the countries afferent to the Mediterranean basin, the Mediterranean Diet is characterized by some common features: consumption of whole grains, a wide variety of local and seasonal fruits and vegetables, moderate amounts of dairy products, plant-based protein sources, and reduced amounts of saturated fats with olive oil and olives being the main source of fat. In addition, it is advised to reduce red meat, drink a moderate amount of red wine, and to use herbs and spices as a salt replacement [3]. The MD has been the subject of several studies due to its positive impact on health, which is protection against major chronic diseases [4] including cardiovascular disease [5] and some cancers [6]; decreasing the odds of being overweight or obese [7]; positive effects on lipoprotein levels and insulin resistance [8]. In addition to its various health benefits, a review study published in 2013 found a consistent pattern between higher adherence to the MD with better cognitive functions and reduced risk of Alzheimer's disease [9].

There is evidence that adherence to the Mediterranean Diet is decreasing in countries traditionally adopting such dietary habits [10], [11]. Lebanon, a country on the Mediterranean coast and has long been adherent to the MD, is experiencing a shift in food choices away from the MD [12]. A study done among university students found that there is a trend of consuming a more westernized diet, especially among males [13] and therefore “encouraging for a new dietary pattern high in fat, refined sugar, and processed foods and causing a higher prevalence of metabolic diseases” [14].

The adherence to the MD among university students in Lebanon has not been a subject of concern in recent studies, although universities “represent the final opportunity for health and nutritional education of a large number of students from the educator's perspective” [15]. Adherence to the MD among university students has been studied and it was found that 37.5% of Turkish university students had low adherence to the MD [16] compared to 21.8% of Cypriot university students [17]. The adherence to the MD in Lebanon was studied among high school adolescents and it was found that 43% of students had low adherence to the MD [14].

One of the tools to assess the adherence to the MD is the Mediterranean Diet Quality Index for children and adolescents (KIDMED), which was developed and validated by Serra-Majem et al. [18]. To our knowledge, the KIDMED index has been used to test the adherence to the MD among school students and not university students. Hence, the aim of this study is to study the association of adherence to the MD with sociodemographic factors, anthropometric measurements, nutritional knowledge, and academic achievement among a representative sample of university students in Lebanon.

## 2. Materials and Methods

### 2.1. Study Design

A cross-sectional study was carried out among selected public and private university students in Lebanon. The top 10 universities in Lebanon were chosen based on the number of registered students according to the report by the Ministry of Education and Higher Education for the academic year of 2017-2018 [19].

### 2.2. Sampling Method

Data from the Ministry of Education was used in order to calculate the number of students needed from the major universities in Lebanon to be a representative sample. Then university students were sent a link to a self-administered online questionnaire. The students had to be currently enrolled in the university and aged below 25 years. A total of 303 students completed the questionnaire and were within the set criteria after excluding incomplete questionnaires and students aged above 25 years (127 students).

### 2.3. Ethical Considerations

The questionnaire used in this study was approved by the Ethics Committee of Holy Spirit University of Kaslik (USEK). A consent form was written at the beginning of the questionnaire for all participants to read and agree to before filling out the questionnaire.

### 2.4. Questionnaire

The questionnaire was composed of 38 questions divided into 5 main sections.

#### 2.4.1. Section 1

This section included questions related to sociodemographic factors and academic achievement, namely gender, age, university, major, level of education, most recent cumulative Grade Point Average (GPA), area of residence, living status, working status, and average monthly income.

Students were considered having excellent academic performance if their most recent GPA score was higher than 3.67 (or higher than 85), good performance if GPA was between 2.67 and 3.67 (or between 76 and 85), average performance if GPA between 1.67 and 2.67 (or between 65 and 75), and poor performance if lower than 1.67 (or lower than 65) [20].

#### 2.4.2. Section 2

In this section of the questionnaire, the students self-administered their weight in kilograms and height in meter and, subsequently, the Body Mass Index (BMI) was calculated by dividing weight in kilograms (kg) by the square of height in meters (m). The BMI was evaluated according to standards put by the World Health Organization (WHO), where a person is considered overweight if BMI ≥ 25 kg/m^2^ and obese if BMI ≥ 30 kg/m^2^ [21]. In addition, the participants were asked to choose from a variety of body pictures for males and females in order to assume their approximate waist/hip ratio.

#### 2.4.3. Section 3

This section of the questionnaire included 4 multiple choice nutritional knowledge questions regarding choosing the healthiest breakfast choice, why it is recommended to have a healthy breakfast, the composition of a healthy meal, and the main components of the MD.

#### 2.4.4. Section 4

In this part of the questionnaire, the subjects were asked about their dietary habits. The questions were if and what meals are usually skipped, how often breakfast is consumed, and reasons why one might consume or skip breakfast.

#### 2.4.5. Section 5

In the final part of the questionnaire, the adherence to the MD was evaluated using the KIDMED test. It is based on a self-administered 16-question test with scores ranging from 0 to 12. Answers with a negative connotation are given a score of −1, and answers with a positive connotation are given a score of +1. Individual scores are added and those having ≥8 are said to have high adherence to the MD, 4–7 medium adherence, and ≤3 low adherence, and therefore, low diet quality [22].

### 2.5. Statistical Analysis

All tests performed were evaluated using Statistical Package for Social Sciences (SPSS) for Windows version 25 (SPSS Inc., Chicago, IL, USA). Qualitative data were described using the number and percent. The associations were calculated on a 95% confidence interval and the significance value to measure the strength of evidence was set at *p* < 0.05.

## 3. Results

### 3.1. Population Characteristics

Of the 303 participants who completed the questionnaire, 210 were females and 93 were males. The majority of the females (56.5%) and males (62.6%) were aged between 21 and 25 years. 58.1% of females and 62.4% of males were undergraduates, and 70.5% of females were unemployed, either currently looking for work or not similar to 69.9% of males. 106 of the participants were students in the Lebanese university, while 197 were in private universities. There was a significant difference (*p* = 0.007) between females and males regarding GPA scores where 81.5% of females had excellent (26.2%) and good performance (55.3%) compared to 63.4% of males with 20.4% having excellent performance and 43.0% having a good performance.

### 3.2. Anthropometric Measurements

The average calculated BMI of females was 22.96 kg/m^2^ ± 3.68 which was significantly lower (*p* = 0.0001) than the average BMI of males which was 25.59 kg/m^2^ ± 4.59. There was a significant difference (*p* = 0.029) between the average BMI of undergraduates (24.29 kg/m^2^ ± 4.26) and graduates 22.86 kg/m^2^ ± 3.55. In addition, there was a significant difference (*p* = 0.033) in the average BMI between students with satisfactory academic performance (24.98 kg/m^2^ ± 4.82) and those with good academic performance (23.30 kg/m^2^ ± 3.47). Regarding the knowledge questions, there was a significant difference (*p* = 0.0001) in the average BMI on answers to the third question regarding components of a healthy meal. Students with correct answers had an average BMI of 22.28 kg/m^2^ ± 3.14 compared to 24.31 kg/m^2^ ± 4.34 of those with false answers. There was also a significant difference (*p* = 0.0001) in the average BMI between correct answers (21.37 kg/m^2^ ± 2.94) and false answers (24.16 kg/m^2^ ± 4.19) on the fourth question regarding knowledge about the characteristics of the MD.

### 3.3. Dietary Habits and Adherence to the MD

The vast majority (79.20%) of the students reported that they always or sometimes skip a meal per day, with breakfast reported to be the most skipped meal. Only 38.28% of the students reported having breakfast every day. When students who sometimes (<7 days/week) or always (0 days/week) skipped breakfast were asked the reason behind not consuming breakfast, the main answer was the lack of time (25.31%) followed by oversleeping (20.04%) and lack of appetite (18.78%). The students who always (7 days/week) or sometimes (<7 days/week) consume breakfast reported that the main reason behind consuming breakfast was to gain energy (30.11%) followed by waking up hungry (26.47%) and to be healthy (20.13%).

Regarding the adherence to the MD, 32.70% of the students had a poor adherence scoring ≤3 on the KIDMED test, 48.50% had an average adherence scoring between 4 and 7, while 18.50% had a high adherence scoring ≥8. As shown in Table 1, there is a significant difference (*p* < 0.0001) between skipping meals and adherence to the MD where the vast majority of students who were with poor adherence to the MD reported to sometimes or always skip a meal (90.9%) compared to 50.88% of those with high adherence. In addition, there was also a significant difference (*p* < 0.0001) between breakfast consumption and adherence to the MD. In the poor adherence to the MD category, only 15.15% of students reported consuming breakfast on a regular basis compared to 71.93% of students who have a high adherence.

Table 2 shows the difference in the average KIDMED score between correct and false answers on the nutritional knowledge questions. There was a significant difference between the average KIDMED scores between correct and false answers on the question related to choosing the healthiest breakfast option, components of a healthy meal, and characteristics of the MD, where in the 3 questions, those with correct answers had a higher average score on the KIDMED test compared to false answers.

**Figure 1:**
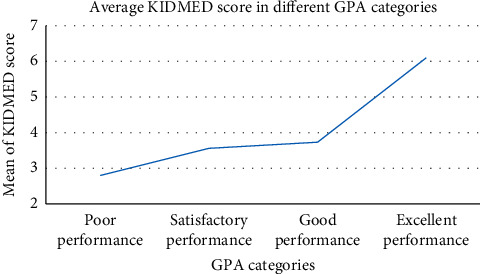
The average of KIDMED score of students in poor and high adherence to MD categories with respect to GPA categories. GPA: Grade Point Average.

Table 1 also shows that there is a significant difference in academic achievement when comparing the students' adherence to the MD. Only 17.17% of students with poor adherence to the MD had excellent GPA scores compared to 45.61% of excellent GPA scores in the high adherence to the MD group. To further investigate the relation between GPA scores and adherence to the MD, only the poor and high adherence to the MD was taken into consideration.  Figure 1 shows the average of the KIDMED scores compared to the academic performance represented by GPA categories of students with poor adherence to the MD compared to those with high adherence. There is a significant difference between the groups (*p* = 0.002), where students with excellent performance had an average of 6.10 ± 3.666, good performance an average of 3.73 ± 3.365, satisfactory performance average of 3.56 ± 3.342, and poor performance an average of 2.80 ± 3.114.

## 4. Discussion

Since the university is considered the last step to engrave mass awareness including nutrition, to a large number of people, good nutrition awareness is needed in order to ensure healthy choices and behaviors in the coming generations. For this reason, this study was done in order to evaluate the level of adherence of university students in Lebanon to the MD and its association with sociodemographic, nutritional knowledge, eating behaviors, and academic achievement.

Similar to a study done among university students in Lebanon in 2014, our study found that males had a higher average BMI than females. The previous study recruited 3384 students from private and public universities and found that a higher percentage of males were overweight or obese due to the fact that they were more prone to choose a westernized diet than females [13]. When comparing both genders as regards to GPA scores, females seem to have higher GPA scores than males. In this study, the GPA scores were self-administered and may be prone to bias. In a study done among undergraduate university students in the United States, a significant difference between genders was found within the lower academic performance group; females in the study tended to overreport their actual GPA while males underreport their actual GPA scores [23].

In this study, students with lower average BMI had significantly more correct answers on the nutritional knowledge questions than those with higher average BMI. In addition to the association between nutritional knowledge and lower BMI, an association was found between higher adherence to the MD and higher nutritional knowledge. These results are consistent with a study done in Italy, which stated that the higher the nutritional knowledge a person has may lead to healthier food choices in addition to reduced rates of obesity [24]. This is an important indicator of the importance of nutrition awareness, especially when it comes to highlighting the importance of the MD and its various health benefits among university students. This will help them have healthier choices and may lead to improved health behaviors.

When students were asked what the most skipped meal was, breakfast was their first choice. The choice for breakfast being the most skipped meal by university students is consistent with studies done in Kuala Lumpur [25] and Turkey [16]. In both of these studies, the most common reason behind skipping breakfast was the lack of time, which was also the major reason in this study. This raises a question regarding the schedule of university students, which is sometimes hectic. Therefore, awareness should be focused on time-management in relation to meal planning. Regarding the reason behind consuming breakfast, the most common answer was to gain energy, which is also consistent with a study done in Egypt with gaining energy in the morning being the primary reason by university students to consume breakfast [26].

This study found a significant association between breakfast consumption and skipping meals with adherence to the MD. This association was established after a previous study done among Lebanese adolescents found that students who tend to skip breakfast had low adherence to the MD compared to students with regular breakfast consumption who all had high adherence [14]. Their study also concluded that not only consuming breakfast is associated with adherence to the MD, but the quality of food eaten at breakfast was also a strong predictor of the adherence to the MD.

Regarding the adherence to the MD, only 18.5% of the students had a high adherence to the MD based on the KIDMED score. When comparing the results to studies done abroad, the numbers in this study are consistent with the study done in Turkey among university students where 17.8% of students had high adherence to the MD [16], but are different than the study done among the university students in Cyprus where 26.9% had high adherence [17]. Although to our knowledge, no previous study was done in Lebanon to evaluate the level of adherence to the MD among university students, the adherence was evaluated among Lebanese high school adolescents and similar to our study, only 15.8% of the participants had high adherence [14]. Since the numbers are consistent among different age groups, this seems to be a trend of shifting dietary habits and straying away from the traditional MD in Lebanon, which raises a concern regarding future dietary habits and choices.

This study found no difference in adherence to the MD between genders, although several previous studies, one of which was done in the Balearic Islands, found that adolescent boys had a higher risk of having low adherence to the MD than girls [27]. This was explained by the fact that girls are more interested in their body image than boys, but this association was not established in our study. However, other studies conducted in the Mediterranean area were in line with our findings and did not find significant differences between boys and girls [28], [29].

The most intriguing association found in this study was the relation between adherence to the MD and academic achievement. This study found that the higher the adherence to the MD, the higher the GPA scores of the students. Although a causal association cannot be established given the nature of the study, nevertheless, this is an important finding. The study was done in Turkey also had similar results where the higher the adherence to the MD, the higher the GPA scores [16]. In a review study published in 2017, it was found that in the majority of studies reviewed, there is a statistically significant positive association between dietary intake/behavior and academic achievement [30]. This can be a leading slogan when advertising for healthy eating, especially among university students, when they learn the importance of healthy eating, namely the MD, and its effect on health in general and its relation with higher GPA scores.

Although to our knowledge, this is one of the first studies in Lebanon to evaluate the association between adherence to the MD and several factors among university students in Lebanon, few limitations cannot be excluded. One of which is that bias cannot be excluded especially since all the answers especially those regarding weight, height, and GPA scores, are self-reported. In addition, a causal link cannot be established given the cross-sectional nature of the study and therefore, further cohort prospective studies need to be done to ensure if a causal link can be established between the variables. Selection bias cannot be excluded since a link to the online questionnaire was disseminated through students and university staff of personal knowledge and therefore, respondents may not represent the whole population of the university students in Lebanon.

## 5. Conclusion

This study found that there is a significant association between adherence to the MD with nutritional knowledge and academic achievement; university students in Lebanon with higher adherence to the MD had higher nutritional knowledge and higher academic achievement. Only 18.8% of students had high adherence to the MD. In addition, breakfast was the most skipped meal, with the lack of time being the main reason. Our results are baseline due to the study's observational nature but open the floor to future nutrition interventions on the university level, which may be the last chance to engrave healthy eating behaviors on a large scale.

## Figures and Tables

**Table 1:** Association between sociodemographic, eating habits, anthropometric characteristics, and adherence to the MD among university students in Lebanon, *N* = 303.

Variables	Adherence to the MD			*p* value
	Poor *n* (%)	Average *n* (%)	High *n* (%)	
Gender				
Females	66 (66.67)	100 (68.03)	44 (77.19)	0.349
Males	33 (33.33)	47 (31.97)	13 (22.81)	

Age				
Between 18 and 21 years	45 (45.45)	60 (41.67)	20 (35.09)	0.449
Between 21 and 25 years	54 (54.55)	84 (58.33)	37 (64.91)	

Level of education				
Undergraduate	62 (65.26)	90 (63.83)	28 (51.85)	0.353
Graduate	25 (26.32)	44 (31.21)	21 (38.89)	
Postgraduate	8 (8.42)	7 (4.96)	5 (9.26)	

GPA categories				
Poor performance	4 (4.04)	6 (4.08)	1 (1.75)	0.019^*∗*^
Satisfactory performance	26 (26.26)	26 (17.69)	10 (17.54)	
Good performance	52 (52.53)	82 (55.78)	22 (38.60)	
Excellent performance	17 (17.17)	33 (22.45)	24 (45.61)	

Employment status				
Employed full-time	13 (13.13)	20 (13.61)	9 (15.79)	0.964
Employed part-time	14 (14.14)	23 (15.65)	11 (19.30)	
Unemployed and currently looking for work	33 (33.33)	49 (33.33)	19 (33.33)	
Unemployed and currently not looking for work	39 (39.40)	55 (37.41)	18 (31.58)	

Average monthly income				
Less than 675,000 LBP	56 (56.57)	85 (57.83)	29 (50.88)	0.797
Between 675,000 LBP and 1,500,000 LBP	28 (28.28)	38 (25.85)	14 (24.56)	
Between 1,500,000 LBP and 3,000,000 LBP	11 (11.11)	15 (10.20)	9 (15.79)	
More than 3,000,000 LBP	4 (4.04)	9 (6.12)	5 (8.77)	

Skipping meals				
Yes	32 (32.32)	28 (19.05)	3 (5.26)	<0.0001^*∗*^
No	9 (9.10)	42 (28.57)	28 (49.12)	
Sometimes	58 (58.58)	77 (52.38)	26 (45.62)	

Breakfast consumption				
Always	15 (15.15)	60 (40.82)	41 (71.93)	<0.0001^*∗*^
Sometimes	74 (74.75)	81 (55.10)	16 (28.07)	
Never	10 (10.10)	16 (10.88)	0 (0)	

BMI categories				
Underweight	6 (6.10)	8 (5.40)	5 (8.80)	0.806
Healthy weight	62 (62.60)	85 (57.80)	37 (64.90)	
Overweight	22 (22.20)	39 (26.50)	12 (21.10)	
Obese	9 (9.10)	15 (10.2)	3 (5.30)	

*p* value: Pearson's Chi-square test and Fisher exact test with more than 20% of expected counts less than 5. ^*∗*^*p* value <0.05 is considered as significant. MD: Mediterranean Diet; GPA: Grade Point Average; BMI: Body Mass Index; LBP: Lebanese Pound.

**Table 2:** Association between answers on nutritional knowledge questions and average KIDMED score among university students in Lebanon.

	*N*	Average KIDMED score	Std. deviation	*p* value
Knowledge regarding healthiest breakfast option				
Correct	223	5.08	2.729	0.014^*∗*^
False	80	4.20	2.650	

Knowledge regarding reasons to consume a healthy breakfast				
Correct	68	5.09	2.532	0.405
False	235	4.77	2.788	

Knowledge regarding components of a healthy meal				
Correct	80	6.05	2.530	<0.0001^*∗*^
False	223	4.41	2.676	

Knowledge regarding characteristics of the mediterranean diet				
Correct	38	5.97	2.765	0.006^*∗*^
False	265	4.68	2.694	

*p* value: Independent sample *t*-test to check if there exists a significant difference between two qualitative and quantitative groups. ^*∗*^*p* value <0.05 is considered as significant.

## Data Availability

The questionnaire, data collected, and SPSS analysis used to support the findings of this study are available from the corresponding author upon request.
